# Balloon Passage via Microwire Navigation Technology for Severe Stenosis of Middle Cerebral Artery: A Case Report

**DOI:** 10.1002/ccr3.73148

**Published:** 2026-08-05

**Authors:** Yang Gao, Puyuan Zhao, Chen Li, Qiang Xie, Yijin Xiang, Zhang Shi, Kai Hou, Xu Liu, Bagus Dermawan, Derfiani Prinanda Hussein, Zhigang Yang

**Affiliations:** ^1^ Department of Neurosurgery Zhongshan Hospital, Fudan University Shanghai China; ^2^ Department of Neurosurgery National Clinical Research Center for Interventional Medicine of China Shanghai China; ^3^ Department of Integrative Medicine Zhongshan Hospital, Fudan University Shanghai China; ^4^ Department of Radiology Zhongshan Hospital, Fudan University Shanghai China; ^5^ Department of Neurology Zhongshan Hospital, Fudan University Shanghai China; ^6^ Department of Neurology Kariadi General Hospital Semarang Central Java Indonesia; ^7^ Department of Neurology Kembangan District Hospital Jakarta Indonesia

**Keywords:** balloon passage, high‐resolution magnetic resonance imaging, microwire navigation, middle cerebral artery stenosis, PCSK9 inhibitors

## Abstract

This case presents that balloon passage via microwire navigation technology could be a beneficial option for patients with severe MCA stenosis, with the diameter of the proximal blood vessel in the stenotic segment is < 1.5 mm and there is antegrade blood flow.

## Key Clinical Message

The submaximal balloon angioplasty proved effective for symptomatic intracranial atherosclerotic stenosis (ICAS), but for the culprit vessel < 1.5 mm, the treatments are rarely reported. Here, we report that balloon passage via microwire navigation technology could be a beneficial option for such patient and achieved satisfactory outcomes.

## Graphical abstract

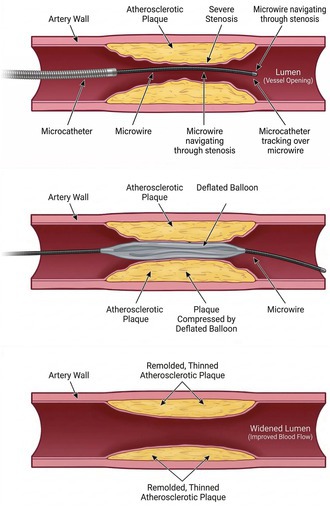

## 1 Introduction

Intracranial atherosclerotic stenosis (ICAS) is one of the most common causes of ischemic stroke worldwide, especially in China, which accounts for up to 46.6% of ischemic stroke and transient ischemic attack (TIA) patients [1, 2]. With the development of endovascular treatment strategy and innovative devices, aggressive revascularization of the qualifying artery has been proven to be effective for elevating favorable outcomes. Specially, the submaximal balloon angioplasty represents a safe and effective method for cerebrovascular stenosis. The submaximal angiography indicated the balloon inflation diameter is sized to approximately 50% to 80% of the normal vessel diameter of at least undersized 0.5 mm of the nearby artery diameter [3, 4]. However, in some ischemic stroke patients, the diameter of the qualifying artery was even < 1.5 mm, while still consisting of antegrade blood flow; the smallest balloon is hardly to be inflated. Herein, our case demonstrated one ischemic stroke case caused by severe stenosis of the middle cerebral artery (MCA) with the proximal artery diameter < 1.5 mm and achieved satisfactory results.

## 2 Case Presentation

### 2.1 Case History and Examination

**FIGURE 1:**
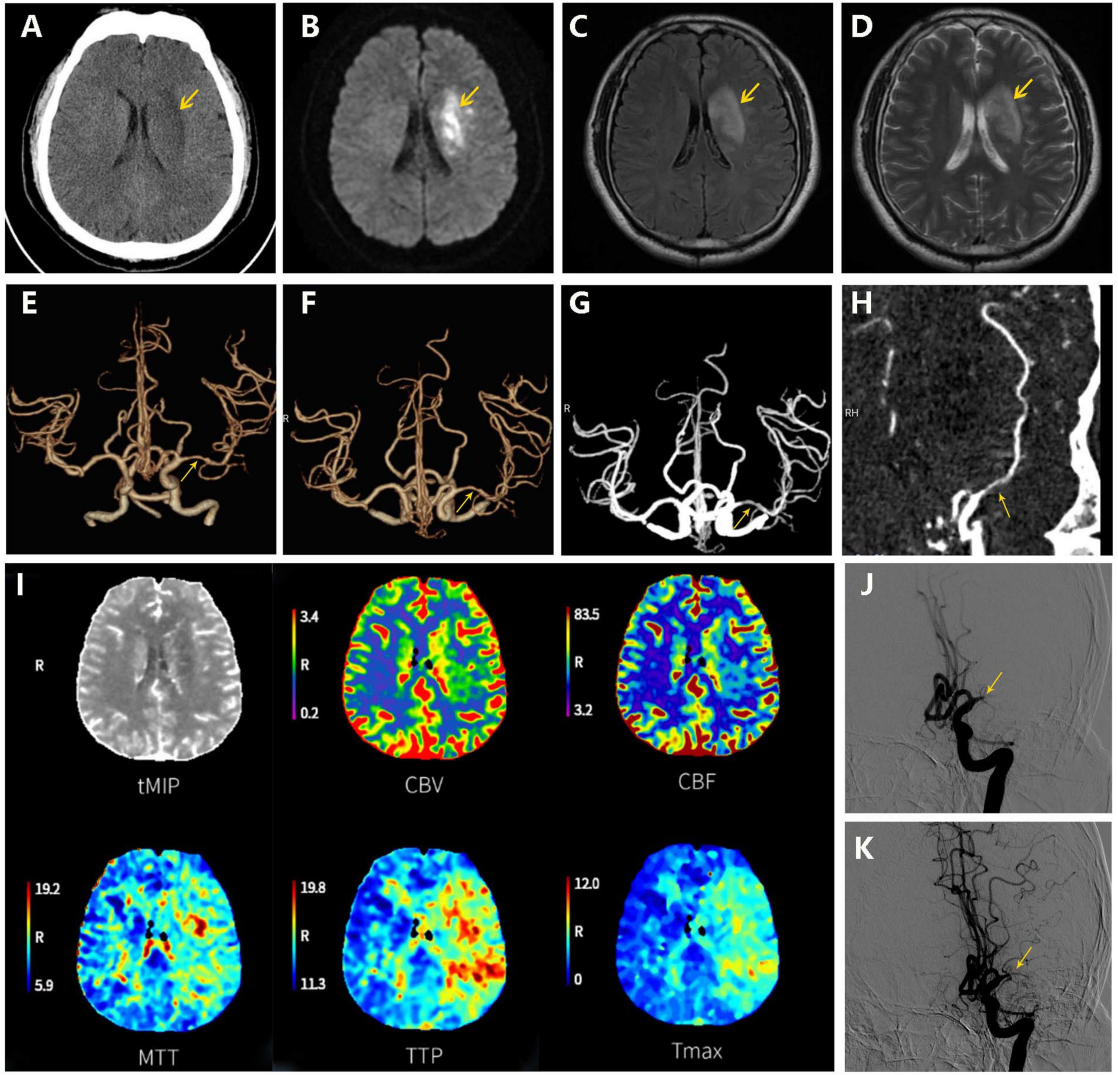
The patient received a series of examinations and was diagnosed as ischemic stroke of left basal ganglia region. (A) Head CT scan shows low density in the left basal ganglia region. (B–D) MRI demonstrated high DWI signal (B), slightly high Flair signal (C), and slightly elevated T2 signal (D) in the left basal ganglia region. (E–H) CTA demonstrated severe stenosis of the left middle cerebral artery (M1) from multiple angles, including 3D reconstruction (E, F), MIP (G), and MPA (H). (I) CTP revealed ischemic changes in the left cerebral hemisphere. (J) Two days later, DSA demonstrated occlusion at the origin of the left middle cerebral artery (J), with distal blood flow supplied via collateral circulation (K).

A 48‐year‐old male patient presented with dizziness, slurred speech, and disappeared right nasolabial folds. The patient was sent to a local hospital and received standardized examinations. Non‐enhanced head CT scan revealed the ischemic stroke of the left basal ganglia region (including caudate head and internal capsule) (Figure 1A), and MRI further confirmed the diagnosis (Figure 1B–D). Then the CT angiography (CTA) and CT perfusion (CTP) revealed severe stenosis of the M1 segment (Figure 1E–H) along with insufficient blood perfusion of the left hemisphere (Figure 1I). The local hospital performed cerebral angiography and found the M1 trunk was occlusive; the distal blood flow was supplied by the collateral branches (Figure 1J,K). Since the patient had an acute stroke, further interventional therapy was not performed due to the high risk of intracranial hemorrhage. Afterwards, he received the best medical treatment, including dual antiplatelet, statin therapy, and blood pressure control.

### 2.2 Investigation and Treatment

**FIGURE 2:**
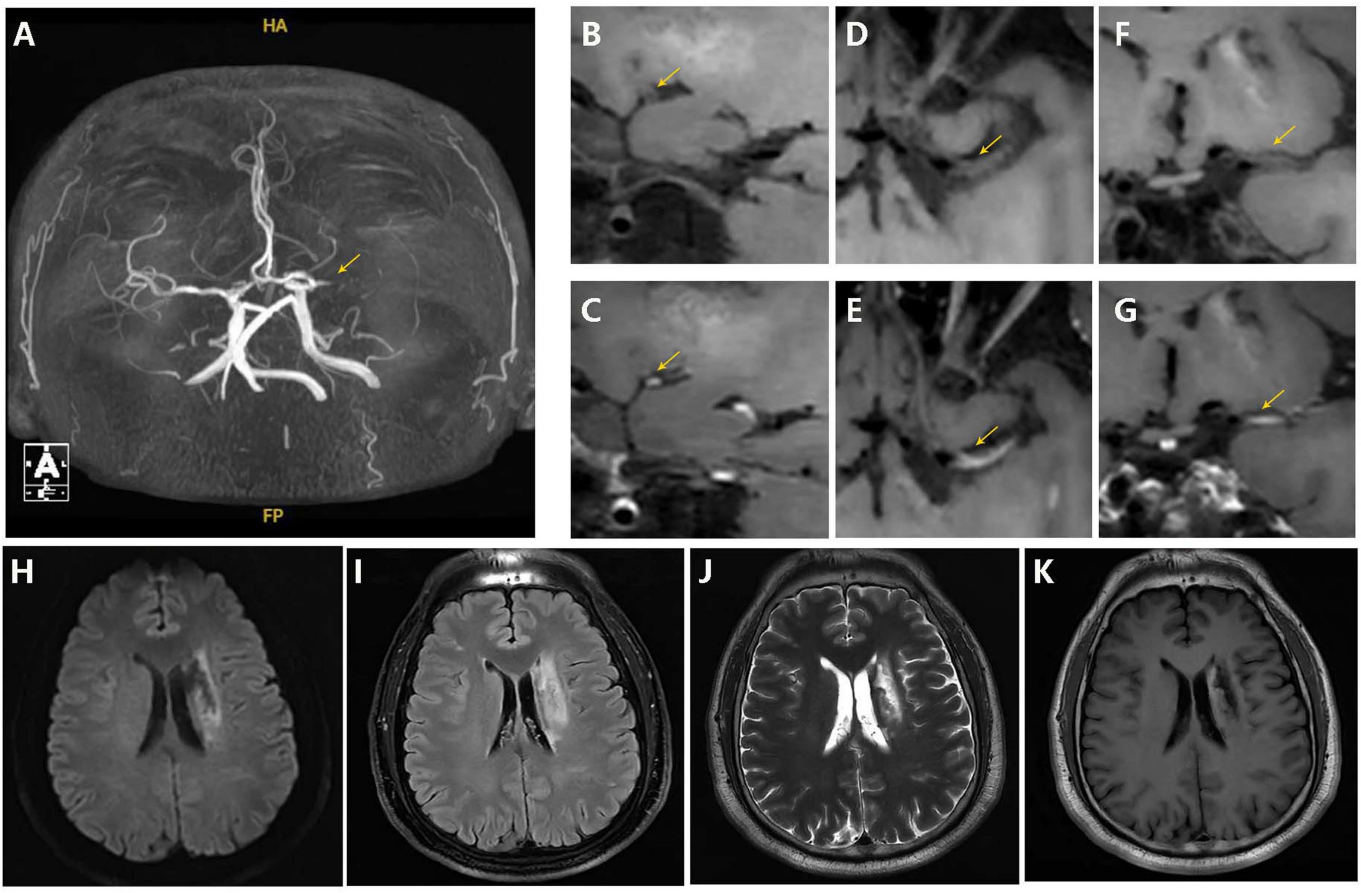
Two months after the onset of the patient's condition, high‐resolution 3 T intravascular wall imaging revealed occlusion in the left M1 segment with no distal blood flow. TOF images (A), MRI sagittal (B), axial (D), and coronal (F) views, as well as enhanced images (C, E, G), demonstrated significant vascular wall enhancement. DWI (H), Flair (I), T2 (J), and T1 sequences (K) on non‐contrast MRI indicated recovery of the left basal ganglia infarct area without evidence of new‐onset cerebral infarction.

Two months later, he came to the outpatient department of Zhongshan Hospital of Fudan University and received three‐dimensional high‐resolution magnetic resonance imaging (HR‐MRI) at 3 Tesla. The results demonstrated severe stenosis of the left M1 segment and no antegrade blood flow appeared (Figure 2A). We also found the enhanced signal of M1 segment after contrast injected (Figure 2B–G). Besides, we also found subacute ischemic stroke of the left basal ganglia region (Figure 2H–K). Then the patient continued to receive aspirin and statin therapy.

**FIGURE 3:**
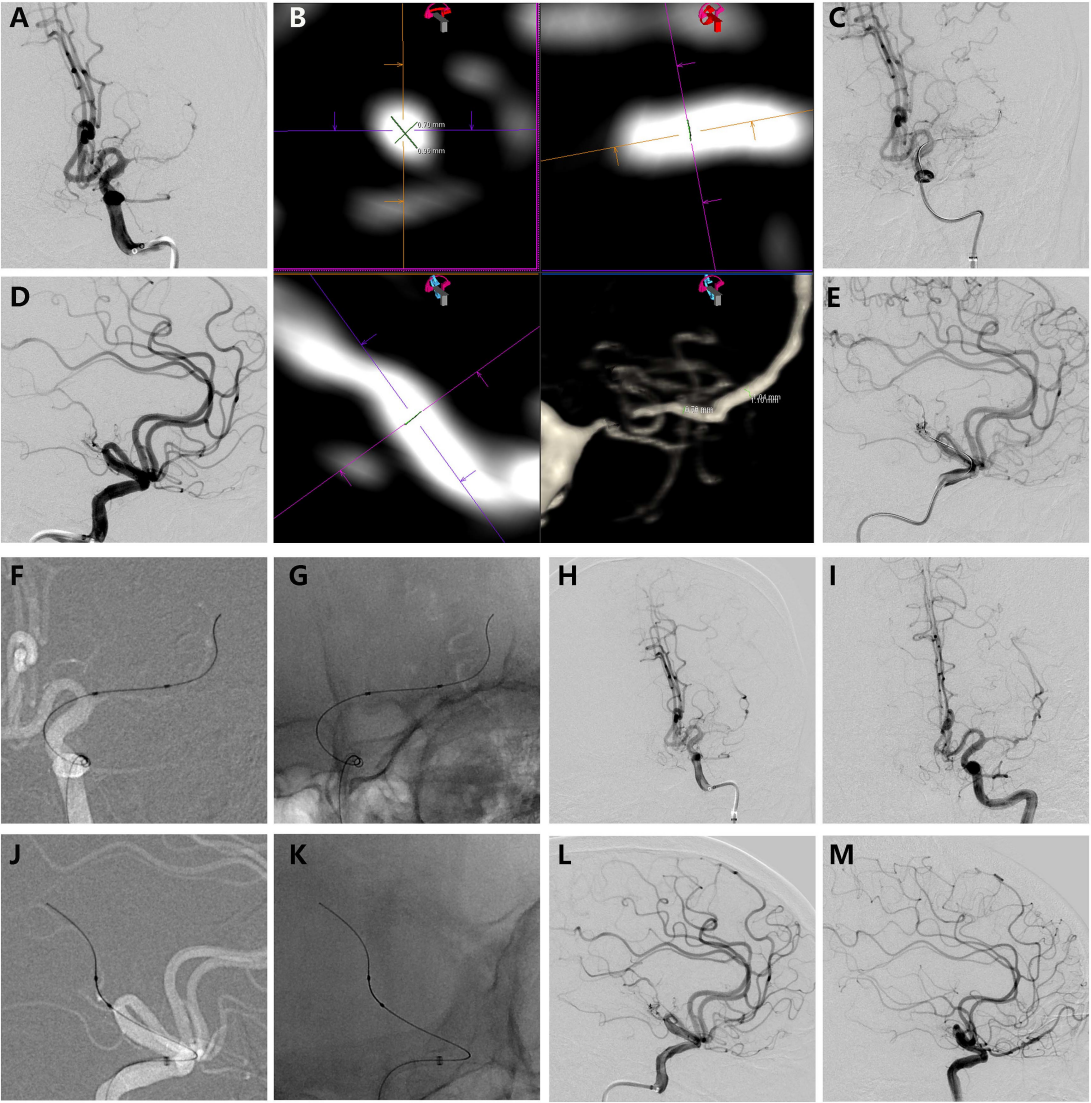
The treatment process of balloon passage via microwire navigation technology of the MCA stenosis. (A, D) working angle angiography revealed severe stenosis of the right middle cerebral artery with antegrade blood flow. (B) eagle‐eye mode measurement showed the maximum diameter of the vessel near the stenosis was only 0.96 mm. (C, E) 200 cm microwire with microcatheter passed through the stenosis. (F–J) exchange 300 cm microwire and balloon reached the stenotic segment. (G, K) balloon passage through the stenotic segment under fluoroscopy. (H, L) Working angle after balloon passage. (I, M) postoperative 6‐month follow‐up angiography images.

**FIGURE 4:**
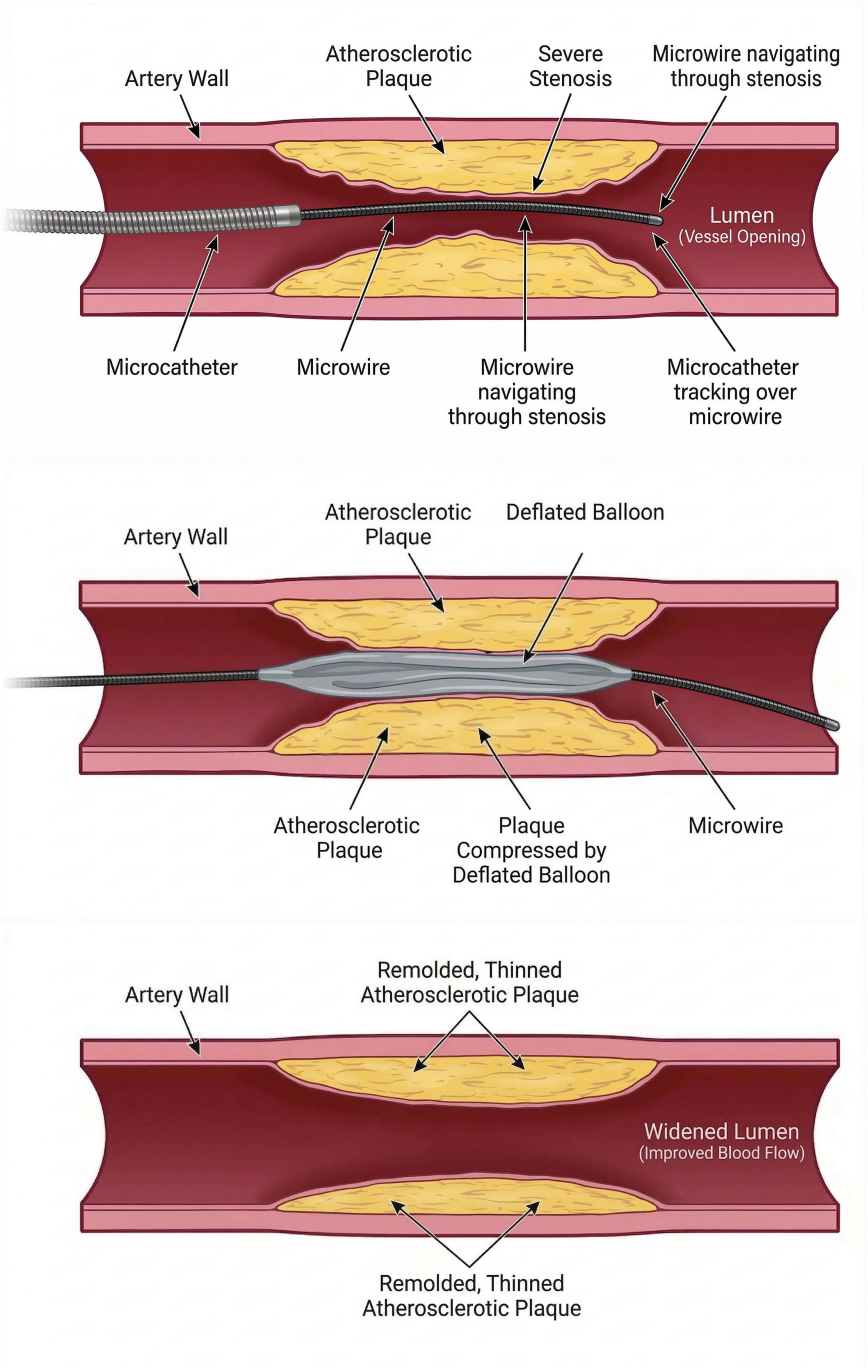
Schematic representation of the balloon passage via microwire navigation technology.

Eleven months later, the patient sought to further aggressive treatment for continuous dizziness and decreased recognition function. He was admitted to the Neurosurgery Department of Zhongshan Hospital. Delicate follow‐up DSA revealed nearly occluded left M1 segment with the existing antegrade blood flow of MCA (Figure 3A,D), but the diameter of the proximal vascular lumen diameter of stenosis is only 0.95 mm (Figure 3B). Considering the insufficient left hemisphere blood perfusion, the patient's relatively young age and his request for preventing potential recurrent stroke, we decided to use the inflatable balloon to traverse the nearly occluded M1 segment. Firstly, the 1.7‐Fr, 150‐cm Echelon 10 microcatheter (Medtronic Corporation) was advanced to the distal position of the severe stenosis assisted with the 0.014‐in. 200 cm Synchro2 microwire (Stryker Corporation) (Figure 3C,E), then the 0.014‐in. 300‐cm Synchro2 microwire (Stryker Corporation) was co‐axis exchanged to the distal MCA (Figure 3F,G,J,K). The rapid exchange 1.5 × 9 mm SacSpeed balloon (Peijia Company) was slowly advanced across the stenosis segment via the 300 cm microwire successfully. Since the vascular diameter of the MCA adjacent to the stenosis was < 1 mm, the inflated balloon could lead to intraoperative vessel damage and devastating hemorrhage, we didn't inflate the balloon but only placed the balloon in the stenosis segment for 3 min and withdrew it subsequently. Then, the final injection demonstrated clearer MCA trunk and its brunch vessels, including the early temporal branch that was visible (Figure 3H,L, Video 1). Figure 4 demonstrates the schematic procedural process of treatment. The patient recovered from interventional treatment uneventfully and received dual antiplatelet treatment (Aspirin 100 mg qd and Clopidogrel 75 mg qd) for 3 months followed by Aspirin (100 mg qd) afterwards and intensified lipid including PCSK9 inhibitor 140 mg every 2 weeks. The patient went to our department for regular follow‐ups.

**VIDEO 1:** Operative procedure of balloon passage via microwire navigation technology and the folllow‐up angiography result. Video content can be viewed at https://onlinelibrary.wiley.com/doi/10.1002/ccr3.73148.

### 2.3 Follow‐Up and Outcomes

**FIGURE 5:**
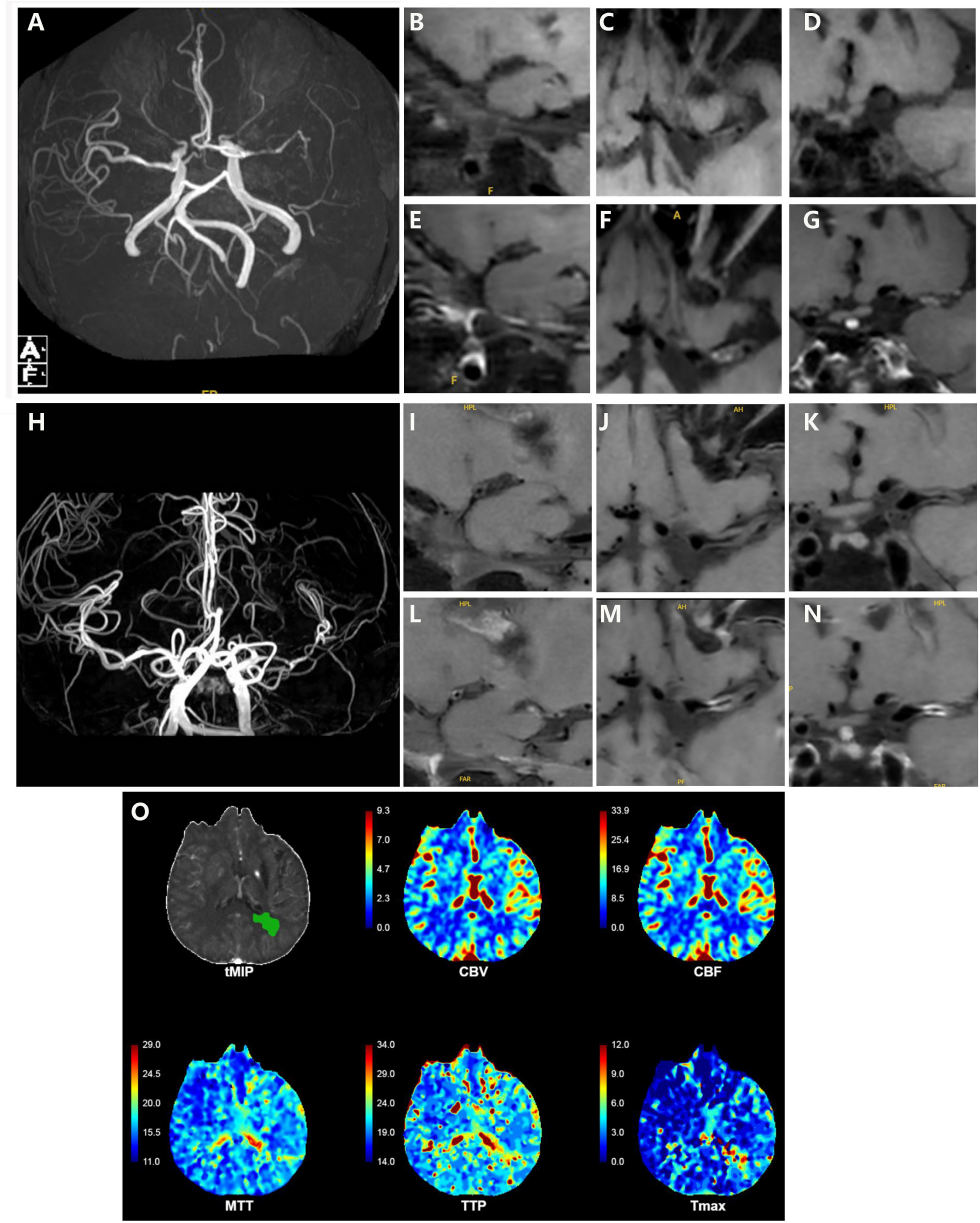
HR‐MRI follow‐ups at 3 T and 5 T, and CTP evaluation 6 months after balloon passage treatment. (A–G) 3 T HR‐MRI images: (A) TOF images; (B–D) sagittal, axial, and coronal non‐contrast images; (E–G), sagittal, axial, and coronal contrast‐enhanced images. (H–N), 5 T HR‐MRI images: (H) 5 T TOF images; (B–D) sagittal, axial, and coronal non‐contrast images; (E–G) sagittal, axial, and coronal contrast‐enhanced images. (O) CTP scan showed significant improvement in left cerebral hemisphere perfusion after 6 months of balloon passage treatment.

The patient was admitted and received regular follow‐up 6 months later. DSA demonstrated that the blood flow of the left MCA was fluent and there was no obvious residual stenosis (Figure 3I,M, Video 1). The HR‐MRI at 3 Tesla also showed the visible early temporal branch (Figure 5A–G). Since the resolution limitation of 3 T HR‐MRI, we also recommend the patient perform 5 T HR‐MRI and the vessel wall could be identified more clearly (Figure 5H–N). The intensity of the vessel wall plaque decreased significantly compared to the last HR examination. We also tested the low‐density lipoprotein cholesterol (LDL‐C) and lipoprotein A (Lp(a)) during the follow‐up period. The patient's LDL‐C level measured 2.58 mmol/L at baseline, which fell to 0.87 mmol/L after 6 months of intervention and consecutive PCSK9 inhibitor administration. Meanwhile, the Lp(a) decreased from 142 to 96 nmol/L. As to the cerebral perfusion, CTP examination also demonstrated elevated blood perfusion in the left hemisphere (Figure 5O).

## 3 Discussion

Submaximal angioplasty has been increasingly demonstrated as a safe and effective treatment for intracranial arterial stenosis, which was defined as dilation to 50%–70% or undersized at least 0.5 mm of the proximal normal vessel diameter [3, 5]. However, in certain special cases, such as symptomatic MCA stenosis with severely reduced proximal vessel diameter (e.g., < 1.5 mm) with antegrade blood flow, the possibility of vessel rupture and hemorrhage will be quite high if we perform submaximal balloon angioplasty. But, the incidence of recurrent stroke within 1 year was as high as 15.1% for symptomatic ICAS despite best medical treatment [6]. In this circumstance, we adopted the balloon passage via microwire navigation technology to expand the vessel lumen and blood flow. We utilized the mechanical force of the balloon and microwire to remove the small plaque. According to Poiseuille's law, blood flow is proportional to the fourth power of the vessel radius; therefore, even a 10% increase in lumen diameter can result in a 46% blood flow increase [4]. At the same time, the leptomeningeal branches of the cerebral hemispheres gradually form compensatory anastomoses, which improve ischemia and alleviate symptoms [7, 8]. To sustain this hemodynamic benefit, we administrated intensive lipid‐lowering therapy after intervention procedure and conducted regular follow‐up evaluations using high‐resolution vessel wall magnetic resonance imaging to evaluate vascular morphology and prognosis. With these comprehensive measures, the patient did not experience new‐onset cerebral infarction events during the 2‐year follow‐up period.

Recent studies have proved that purely balloon angioplasty plus best medical therapy significantly reduces stroke and TIA compared to best medical therapy alone, which provided high‐level evidence to support proactive interventional treatment for intracranial arterial stenosis [3, 5]. When the balloon passes through the stenotic segment of the artery, the mechanical force redistributes the atherosclerotic plaque and disturbs the process of plaque formation, thus expanding the diameter of the artery lumina and improving the blood perfusion [9, 10]. Drug‐coated balloon is a promising alternative for the ICAS, which was transferred to the target lesion and penetrated drugs (eg., paclitaxel or rapamycin) into the vessel wall and inhibited the intimal proliferation and re‐stenosis [11, 12]. However, the long‐term effect of drug‐coated balloon still should be validated by more multicenter randomized clinical trials.

MCA stenosis can lead to stroke mainly through three mechanisms: lenticulostriate perforating vessel occlusion by theombotaeroma, distal vessel occlusion from atheromatous thrombosis, and borderzone infarction caused by insufficient perfusion [13]. In this case, the thalamic infarction was primarily caused by stenosis of the lentiform artery originating from the MCA stem vessels [14]. After balloon passage, no additional mechanical force was imposed, resulting in minimal vascular disturbance and a relatively safe procedure. Additionally, the balloon itself possesses a certain radial tension, which promotes the improvement of the MCA diameter. This procedure requires neurointerventional physicians to maintain high operational stability, avoiding vessel perforation during the procedure to prevent hemorrhage.

In the balloon passage procedure, the balloon was maintained at the target stenotic site for 3 min without inflation. This positioning strategy was adopted for two core reasons: first, sustained gentle mechanical remodeling facilitates redistribution of atherosclerotic plaque and alleviates vascular elastic recoil of the M1 segment; second, slow, protracted balloon retention reduces abrupt stretching of the fragile intracranial vessel wall, lowering risks of arterial dissection and thromboembolism during angioplasty for ICAS. Previous studies on ICAS angioplasty have validated that 2–3 min low‐pressure balloon retention achieves better residual stenosis outcomes without elevating perioperative adverse event rates [5, 15].

Periprocedural management is crucial for successful recovery of ICAS. In addition to the conventional 5–7 days dual antiplatelet therapy, intensive lipid‐lowering therapy is essential for plaque stabilization [16]. Previous studies on proprotein convertase subtilisin/kexin type 9 (PCSK9) inhibitors have demonstrated that their use reduces plaque enhancement, inhibits plaque progression, and plays a significant role in lowering low‐density lipoprotein LDL‐C and Lp(a) levels [17, 18]. Currently, the patient's LDL‐C levels are well‐controlled, which is critical for reducing the risk of cerebral infarction recurrence.

High‐resolution magnetic resonance vessel wall imaging has become increasingly crucial in ischemic cerebrovascular diseases [19]. It enables the assessment of plaque enhancement, presence of atherosclerotic plaque segregation, and vessel wall thickness, all of which play a vital role in guiding intraoperative treatment [20, 21]. For the diagnosis of intracranial aneurysm, 5 T high‐resolution MRI demonstrates further unique advantages in the morphological assessment of distal small cerebral arteries due to its superior spatial resolution [14, 22]. In this case, the patient's thin lumen precluded further balloon dilation. During the procedure, we adopted a strategy of visualizing the vessel without performing balloon expansion. Follow‐up imaging revealed that 5 T HR‐MRI provided clearer visualization of vascular structures compared to 3 T HR‐MRI, highlighting the potential value of high‐resolution 5 T HR‐MRI in cerebrovascular interventional therapy [14, 23].

In summary, for patients with severe MCA stenosis where the diameter of the proximal blood vessel in the stenotic segment is < 1.5 mm and there is antegrade blood flow, treatment with simple balloon passage combined with intensive lipid‐lowering, control of blood pressure, blood glucose, and other risk factors is beneficial for improving cerebral perfusion, reducing the occurrence of restenosis, decreasing the incidence of stroke and TIA, and improving patients' quality of life.

## 4 Conclusion

For patients with severe MCA stenosis where the diameter of the proximal blood vessel in the stenotic segment is < 1.5 mm and there is antegrade blood flow, treatment with simple balloon passage combined with intensive lipid‐lowering, control of blood pressure, blood glucose, and other risk factors is beneficial for improving cerebral perfusion, reducing the occurrence of restenosis, decreasing the incidence of stroke and TIA, and improving patients' quality of life.

## Author Contributions

**Yang Gao:** conceptualization, data curation, formal analysis, investigation, methodology, resources, software, writing – original draft, writing – review and editing. **Puyuan Zhao:** conceptualization, formal analysis, investigation, methodology, project administration, software, supervision, validation. **Chen Li:** data curation, formal analysis, investigation, methodology, resources, software. **Qiang Xie:** conceptualization, data curation, formal analysis, investigation. **Yijin Xiang:** investigation, methodology, resources, software, validation, visualization. **Zhang Shi:** investigation, methodology, funding acquisition, resources, software, visualization. **Kai Hou:** investigation, methodology, resources, software, validation, visualization. **Xu Liu:** investigation, methodology, resources, visualization. **Bagus Dermawan:** formal analysis, investigation, methodology, resources. **Derfiani Prinanda Hussein:** formal analysis, investigation, methodology, resources. **Zhigang Yang:** conceptualization, formal analysis, funding acquisition, investigation, methodology, project administration, resources, supervision, validation, visualization, writing – original draft, writing – review and editing.

## Funding

This work was supported by the National Natural Science Foundation of China (No. 82202145), Shanghai Municipal Health Commission, and Shanghai Administration of Traditional Chinese Medicine (ZXXT‐202310).

## Consent

Written informed consent for publication of the clinical details and images was obtained from the patient. A copy of the consent is available for review by the journal editor upon request.

## Conflicts of Interest

The authors declare no conflicts of interest.

## Data Availability

The data that support the findings of this study are available from the corresponding author upon reasonable request.

## References

[1] Y. Wang , X. Zhao , L. Liu , et al., “Prevalence and Outcomes of Symptomatic Intracranial Large Artery Stenoses and Occlusions in China: The Chinese Intracranial Atherosclerosis (CICAS) Study,” Stroke 45, no. 3 (2014): 663–669. 24481975 10.1161/STROKEAHA.113.003508

[2] T. N. Turan , O. O. Zaidat , G. S. Gronseth , et al., “Stroke Prevention in Symptomatic Large Artery Intracranial Atherosclerosis Practice Advisory: Report of the AAN Guideline Subcommittee,” Neurology 98, no. 12 (2022): 486–498. 35314513 10.1212/WNL.0000000000200030 PMC8967328

[3] C. J. Stapleton , Y. F. Chen , H. Shallwani , et al., “Submaximal Angioplasty for Symptomatic Intracranial Atherosclerotic Disease: A Meta‐Analysis of Peri‐Procedural and Long‐Term Risk,” Neurosurgery 86, no. 6 (2020): 755–762. 31435656 10.1093/neuros/nyz337 PMC7534488

[4] S. M. Seyedsaadat , Y. U. Yolcu , A. Neuhaus , et al., “Submaximal Angioplasty in the Treatment of Patients With Symptomatic ICAD: A Systematic Review and Meta‐Analysis,” Journal of NeuroInterventional Surgery 12, no. 4 (2020): 380–385. 31748381 10.1136/neurintsurg-2019-015451

[5] X. Sun , Y. Deng , Y. Zhang , et al., “Balloon Angioplasty vs Medical Management for Intracranial Artery Stenosis: The BASIS Randomized Clinical Trial,” JAMA 332, no. 13 (2024): 1059–1069. 39235816 10.1001/jama.2024.12829 PMC11378071

[6] O. O. Zaidat , B. F. Fitzsimmons , B. K. Woodward , et al., “Effect of a Balloon‐Expandable Intracranial Stent vs Medical Therapy on Risk of Stroke in Patients With Symptomatic Intracranial Stenosis: The VISSIT Randomized Clinical Trial,” JAMA 313, no. 12 (2015): 1240–1248. 25803346 10.1001/jama.2015.1693

[7] S. M. Uniken Venema , J. W. Dankbaar , A. van der Lugt , D. W. J. Dippel , and H. B. van der Worp , “Cerebral Collateral Circulation in the Era of Reperfusion Therapies for Acute Ischemic Stroke,” Stroke 53, no. 10 (2022): 3222–3234. 35938420 10.1161/STROKEAHA.121.037869

[8] N. F. Binder , M. El Amki , C. Gluck , et al., “Leptomeningeal Collaterals Regulate Reperfusion in Ischemic Stroke and Rescue the Brain From Futile Recanalization,” Neuron 112, no. 9 (2024): 1456–1472. 38412858 10.1016/j.neuron.2024.01.031

[9] J. Gutierrez , T. N. Turan , B. L. Hoh , and M. I. Chimowitz , “Intracranial Atherosclerotic Stenosis: Risk Factors, Diagnosis, and Treatment,” Lancet Neurology 21, no. 4 (2022): 355–368. 35143758 10.1016/S1474-4422(21)00376-8

[10] L. G. S. Almeida , G. Semione , L. T. A. Carretta , et al., “Drug‐Coated Balloon Versus Stent Angioplasty in Patients With Intracranial Atherosclerotic Disease: A Systematic Review and Meta‐Analysis,” Neurosurgical Review 48, no. 1 (2025): 480. 40465011 10.1007/s10143-025-03630-x

[11] B. Li , Q. Bian , H. Li , et al., “Effect of Drug‐Coated Balloon Versus Stent Angioplasty in Patients With Symptomatic Intracranial Atherosclerotic Stenosis,” Operative Neurosurgery 27, no. 6 (2024): 730–738. 38781497 10.1227/ons.0000000000001200 PMC11554355

[12] L. Sun , C. Chen , W. Zhao , et al., “Drug‐Coated Balloon Versus Conventional Balloon for Stent Angioplasty in Symptomatic Intracranial Atherosclerotic Stenosis,” Frontiers in Neurology 16 (2025): 1686858. 41573401 10.3389/fneur.2025.1686858 PMC12819204

[13] T. Ueda , T. Takada , S. Nogoshi , T. Yoshie , S. Takaishi , and T. Fukano , “Long‐Term Outcome of Balloon Angioplasty Without Stenting for Symptomatic Middle Cerebral Artery Stenosis,” Journal of Stroke and Cerebrovascular Diseases 27, no. 7 (2018): 1870–1877. 29530461 10.1016/j.jstrokecerebrovasdis.2018.02.019

[14] Z. Shi , X. Zhao , S. Zhu , et al., “Time‐Of‐Flight Intracranial MRA at 3 T Versus 5 T Versus 7 T: Visualization of Distal Small Cerebral Arteries,” Radiology 306, no. 1 (2023): 207–217. 36040333 10.1148/radiol.220114

[15] T. M. Dumont , A. Sonig , M. Mokin , et al., “Submaximal Angioplasty for Symptomatic Intracranial Atherosclerosis: A Prospective Phase I Study,” Journal of Neurosurgery 125, no. 4 (2016): 964–971. 26745485 10.3171/2015.8.JNS15791

[16] J. W. Chung , J. Cha , M. J. Lee , et al., “Intensive Statin Treatment in Acute Ischaemic Stroke Patients With Intracranial Atherosclerosis: A High‐Resolution Magnetic Resonance Imaging Study (STAMINA‐MRI Study),” Journal of Neurology, Neurosurgery, and Psychiatry 91, no. 2 (2020): 204–211. 31371644 10.1136/jnnp-2019-320893

[17] W. Zeng , F. Zhou , H. Zhao , et al., “Evaluation of Intensive Statins and Proprotein Convertase Subtilisin/Kexin Type 9 Inhibitors on Intracranial Artery Plaque Stability: A Prospective Single‐Arm Study,” Journal of the American Heart Association 14, no. 2 (2025): e035651. 39818872 10.1161/JAHA.124.035651 PMC12054507

[18] W. Tian , H. Cao , X. Li , et al., “Adjunctive PCSK9 Inhibitor Evolocumab in the Prevention of Early Neurological Deterioration in Non‐Cardiogenic Acute Ischemic Stroke: A Multicenter, Prospective, Randomized, Open‐Label, Clinical Trial,” CNS Drugs 39, no. 2 (2025): 197–208. 39755915 10.1007/s40263-024-01145-5

[19] S. Sanchez , M. Mossa‐Basha , V. Anagnostakou , D. S. Liebeskind , and E. A. Samaniego , “Comprehensive Imaging Analysis of Intracranial Atherosclerosis,” Journal of NeuroInterventional Surgery 17, no. 3 (2025): 311–320. 38719445 10.1136/jnis-2023-020622

[20] J. Sun , X. R. Feng , P. Y. Feng , et al., “HR‐MRI Findings of Intracranial Artery Stenosis and Distribution of Atherosclerotic Plaques Caused by Different Etiologies,” Neurological Sciences 43, no. 9 (2022): 5421–5430. 35616814 10.1007/s10072-022-06132-6

[21] S. Liu , M. Lu , X. Gao , et al., “The Diagnostic Performance of High‐Resolution Magnetic Resonance‐Vessel Wall Imaging in Differentiating Atherosclerosis‐Associated Moyamoya Vasculopathy From Moyamoya Disease,” European Radiology 33, no. 10 (2023): 6918–6926. 37453985 10.1007/s00330-023-09951-z

[22] E. A. Samaniego , J. A. Roa , and D. Hasan , “Vessel Wall Imaging in Intracranial Aneurysms,” J Neurointerv Surg 11, no. 11 (2019): 1105–1112. 31337731 10.1136/neurintsurg-2019-014938

[23] Z. Wei , Q. Chen , S. Han , et al., “5T Magnetic Resonance Imaging: Radio Frequency Hardware and Initial Brain Imaging,” Quantitative Imaging in Medicine and Surgery 13, no. 5 (2023): 3222–3240. 37179946 10.21037/qims-22-945 PMC10167427

